# An economic model to assess the costs and benefits of workplace mental wellbeing interventions: A flexible tool for employers and decision makers

**DOI:** 10.1371/journal.pmen.0000601

**Published:** 2026-04-29

**Authors:** Karina Watts, Hannah Ross, Emily Gregg, Matthew Taylor

**Affiliations:** York Health Economics Consortium, Enterprise House, Heslington, University of York, York, United Kingdom; Makerere University, UGANDA

## Abstract

Poor mental wellbeing is one of the leading causes of long-term sickness absence from work and may lead to absenteeism, presenteeism and staff turnover, costing UK employers an estimated £51 billion annually. This study uses economic modelling to provide data on costs and benefits to employers who are considering implementing a workplace intervention to improve wellbeing. Additionally, the analysis is used to assess any changes in employee outcomes (e.g., productivity and staff turnover). A cost-consequence model with a one-year time horizon was developed to assess the impact of workplace mental wellbeing interventions. Because all workplaces are different, it is not useful to present one single base case to generalise across all settings. Instead, the model generates a series of hypothetical case studies, with varying levels of absenteeism, presenteeism and staff turnover, as well as different levels of productivity and staff replacement costs. Several mental wellbeing interventions are compared with ‘no intervention’ (current practice) to calculate the total incremental costs and incremental cost per employee. In a hypothetical case study with 50 employees and an intervention cost of £100, the intervention has a net cost saving of £4,207 per employee. Savings are due to reductions in absenteeism and presenteeism. Sensitivity analysis and scenario analysis assess the impact of varying each input, to reflect that inputs will vary substantially for each individual organisation and setting. The intervention is more likely to be cost saving when the baseline levels of absenteeism, presenteeism and staff turnover are high, and the intervention cost is low. Mental wellbeing interventions may influence a range of outcomes, but outcomes demonstrating a mental wellbeing benefit to employees may be challenging to translate into monetary value. The model can be used by decision makers and employers to understand the potential economic and wellbeing implications of implementing workplace mental wellbeing interventions.

## Introduction

Poor mental wellbeing is one of the leading causes of increased presenteeism and long-term sickness absence from work despite conditions, such as stress and anxiety, being treatable and often preventable [1]. In 2016, Chrisholm et al. estimated that productivity losses due to depression and anxiety cost the global economy over US$ 1 trillion each year [2]. Productivity loss is a measurable decline in an organisation’s output [3,4]. Absenteeism refers to productivity loss because of an employee being absent from work due to mental or physical sickness [3,4]. Presenteeism refers to productivity loss because of an employee being present at work but at a reduced capacity due to either mental or physical health problems [3,4].

During the COVID-19 pandemic, the mental health and wellbeing of employees became even more relevant due to social restrictions, financial stress, healthcare treatment disruptions, and new working patterns [5]. Analysis conducted by Deloitte estimated that poor employee mental health from absenteeism, presenteeism and staff turnover had an annual cost to UK employers of £55 billion in 2020/2021 [6]. In 2022/2023, the estimated annual cost was lower at £51 billion (due to a decrease in presenteeism and staff turnover costs), but this still represents a substantial cost to employers [6].

Some organisations have introduced interventions/programmes that aim to promote positive mental wellbeing in the workplace and support employees’ mental health. The programmes are highly varied in both size and scope, but might include examples such as employee assistance programmes, physical health and activity programmes, financial wellbeing programmes and work culture/manager training programmes. A survey by the Chartered Institute of Personnel and Development found that the proportion of organisations with a wellbeing strategy has increased from 2020 to 2025, with 44% and 57% of organisations having a standalone wellbeing strategy, respectively. Another key finding was that workplace mental wellbeing interventions were shown to reduce sickness absence while increasing productivity [7].

As a result, employers could see improved performance and increased profits [8]. Therefore, economic evaluations of workplace mental wellbeing interventions are important to help employers understand whether implementing interventions will be cost effective or cost saving, and to determine which would have the highest return on investment [6]. It has been estimated that by investing in workplace mental health, employers could achieve a return of £4.70 for every £1 invested; however, this may vary depending on the type and nature of the intervention and the workplace [6]. Several systematic reviews of economic evaluations of workplace mental wellbeing interventions have been undertaken [9–11]. These studies found that interventions may be cost effective for employers and society, but due to a limited number of high-quality studies, strong conclusions cannot be drawn. The limited economic evidence of mental wellbeing interventions may deter employers from adopting these interventions [10].

In 2022, the World Health Organization published guidelines on mental health at work that provided recommendations around optimising the implementation of such interventions [12]. These guidelines further highlighted the gap in cost-effectiveness research for mental health interventions. A challenge in addressing this gap is that while interventions can be effective and, in some cases, cost saving, this can vary depending on the specific organisation and intervention in question. Therefore, the objectives of this study were:

- To conduct economic modelling and provide data on costs and benefits to employers who are considering implementing a mental wellbeing intervention in the workplace.
- To provide a flexible model that both employers and decision makers can use to understand the economic and wellbeing implications when considering implementing a new workplace mental wellbeing intervention.

## Materials and methods

A simple cost-consequence model was developed to assess the impact of workplace mental wellbeing interventions from both the employer perspective and a wider perspective, including employee outcomes. The model has previously been used to develop public health guidance issued by the National Institute for Health and Care Excellence [13]. A one-year time horizon was used to reflect relevant effectiveness studies relating to mental health interventions that were generally limited to short time horizons of between 3 and 18 months.

The cost and effectiveness of a mental wellbeing intervention can be affected by many factors. For example, the market sector, employee characteristics, the number of employees receiving the intervention, the type of intervention, and factors external to the workplace. As such, a hypothetical case study is presented for demonstrative purposes, and an interactive model is available online for users to input their own values to generate bespoke results, specific to their workplace.

### Model structure

**Fig 1:**
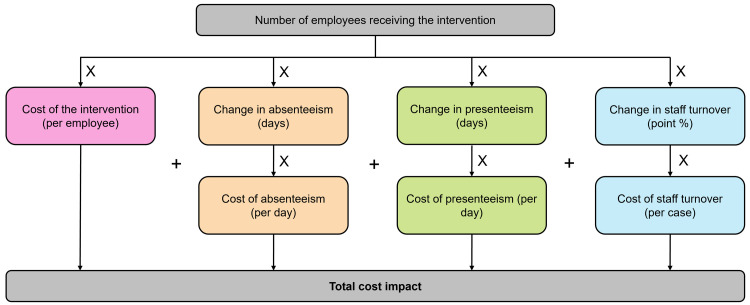
Model structure.

The model structure is shown in Fig 1. The number of employees receiving the intervention was multiplied by each category of the model: the cost of the intervention, the cost of absenteeism, the cost of presenteeism, and the cost of staff turnover. These figures were summed to produce the net cost impact of the intervention. Several mental wellbeing interventions were compared with ‘no intervention’ (current practice) to calculate the total incremental costs and incremental cost per employee. Direct, head-to-head comparisons of different interventions were not possible because of heterogeneity in source data.

### Model inputs

A pragmatic search was conducted to identify baseline costs associated with absenteeism, presenteeism, and staff turnover, as well as effectiveness estimates. Costs were inflated to 2024 prices using the Office for National Statistics Consumer Price Index including owner occupiers’ housing (CPIH) [14].

The reduction in absenteeism is defined as the average number of sick days per year avoided due to the intervention. It is assumed that any reduction in absenteeism is as a direct result of the intervention. The cost of absenteeism (per day) is the estimated cost of a working day, including the cost of wage or salary plus additional costs, such as national insurance and pension contribution. The estimate used in the model was adjusted downwards to take into account that lower-paid workers tend to take more time off work than those who are higher earners (based on evidence given in absence surveys) [15]. The reduction in presenteeism is defined as the average number of lost days due to reduced on-the-job productivity per year avoided due to the intervention. The cost of presenteeism is the estimated cost per working day, including the cost of wage or salary plus additional costs, such as national insurance and pension contribution. However, it is assumed that the cost of presenteeism is higher than the corresponding cost of absenteeism because the inverse association between earnings and rates of sickness absence does not apply to presenteeism [16]. The reduction in staff turnover was not calculated in the hypothetical case study because of a lack of evidence on effectiveness of mental wellbeing interventions.

For the purposes of this analysis, a hypothetical case study is evaluated. The case study does not represent a particular ‘type’ of intervention; it is anticipated that every intervention will differ for different organisations and settings. Such an intervention could be similar to a training programme, a physical exercise programme, a digital platform, or the introduction of flexible work and scheduling changes. The case study is simply to illustrate how the model’s inputs and outputs work. We also undertake evaluations of different scenarios to demonstrate how the results can vary from setting to setting. The inputs for the hypothetical case study used in the base case analysis are presented in Table 1. They combine evidence from a variety of sources and assumptions. Impacts on productivity are based on those reported by Mills et al. (2007) [17], and costs are based on the Centre for Mental Health report (2017) [15]. Intervention costs varied from free (i.e., the intervention was freely available online and employee time was not considered) to £661 per person depending on a range of factors, such as delivery method, length of implementation, and resource usage. The hypothetical case study assumes the cost of the intervention to be £100 per person with 50 employees.

**Table 1:** Hypothetical case study inputs.

Parameter	Inputs	Source
Number of employees	50	Assumption
Cost of the intervention (per employee)	£100	Assumption
**Absenteeism**		
Cost of absenteeism per day	£189.76	Mental health at work: The business cost ten years on (2017) [15] ONS CPIH (2025) [14] Impact of a Health Promotion Program on Employee Health Risks and Work Productivity (2007) [17]
Reduction in absenteeism (in days)	4.3	
**Presenteeism**		
Cost of presenteeism per day	£379.51	Mental health at work: The business cost ten years on (2017) [15] ONS CPIH (2025) [14] Impact of a Health Promotion Program on Employee Health Risks and Work Productivity (2007) [17]
Reduction in presenteeism (in days)	9.6	
**Staff turnover**		
Cost per case of staff turnover	£18,488.43	Mental health at work: The business cost ten years on (2017) [15] ONS CPIH (2025) [14]
Reduction in staff turnover (%)	No evidence	

Abbreviations: ONS, Office for National Statistics; CPIH, Consumer Prices Index including owner occupiers’ housing costs.

To calculate total costs, the cost of the intervention per person was multiplied by the number of participating employees.

To calculate total benefits, the reduction in the number of days of staff presenteeism and absenteeism, and the percentage reduction in staff turnover, were multiplied by their associated unit costs. These were then summed and multiplied by the number of participating employees.

Incremental costs were then derived by deducting total benefits from total costs.

### Sensitivity analysis

One-way sensitivity analysis assessed the impact of varying each input, to reflect that the inputs will vary substantially for each individual organisation and setting [18]. Each input variable was varied independently assuming all other input variables remained the same.

### Scenario analysis

Because all workplaces are different, the hypothetical case study presented in the base case analysis was varied in scenario analyses. The cost of the intervention and number of employees, absenteeism, presenteeism and staff turnover were varied, as well as different levels of productivity and staff replacement costs. The scenarios are presented in Table 2. These scenarios are not based on empirical evidence but rather hypothetical values to explore how the findings of the model might vary across different settings and with different assumptions around inputs. This reflects the fact that all employers and settings are different, and no results from the model will be generalisable to all real-world scenarios. Some measurement instruments are specified as examples of instruments used in real-life studies.

**Table 2:** Hypothetical scenarios used in scenario analysis.

Hypothetical scenarios	Inputs
Scenario 1: Large company with high levels of absenteeism and presenteeism	600 employees £5,000 online stress reduction program Company allows one working day for each employee to engage in program (average cost of £105,000 per year) Cost of absenteeism and presenteeism assumed to be equal at average cost of £216 per day Annual reduction of 1.3 days in absenteeism and 2.5 days in presenteeism
Scenario 2: Medium company with low levels of absenteeism	250 employees £160 per participant for 4 sessions of cognitive behavioural therapy Opt-in approach, 75 employees opted in Average cost of absenteeism was £259 per employee per day Annual reduction of 0.6 days in absenteeism
Scenario 3: Small company with medium levels of absenteeism and staff turnover	100 employees £300 per participant training sessions Average cost of absenteeism was £370 per person per day and average cost per case of staff turnover was £22,799 Annual reduction of 1.1 days in absenteeism and 0.5% annual reduction in staff turnover
Scenario 4: Small company with high levels of staff turnover	40 employees £52 average cost per employee for intervention Average cost per case of staff turnover was £9,119 Annual reduction of 2% in staff turnover
Scenario 5: Micro company with low levels of absenteeism and medium presenteeism	5 employees £165 per participant training sessions Cost of absenteeism and presenteeism assumed to be equal at average cost of £148 per person per day Annual increase of 0.3 days in absenteeism and annual reduction of 1.8 days in presenteeism

Scenario 1 looks at the potential impact of an online stress reduction program on a large-sized company with high levels of absenteeism and presenteeism. Scenario 2 looks at the potential impact of opt-in cognitive behavioural therapy sessions on a medium-sized company with low levels of absenteeism. Scenario 3 looks at the potential impact of participant training sessions on a small-sized company with medium levels of absenteeism and staff turnover. Scenario 4 looks at the potential impact of an intervention on a small-sized company with high levels of staff turnover. Scenario 5 looks at the potential impact of participant training sessions on a micro company with low levels of absenteeism and medium levels of presenteeism. The aim of modelling these various scenarios is to demonstrate the impacts of different available workplace mental health interventions on different company sizes and different levels of staff retention and staff sickness.

## Results

### Hypothetical case study

In the hypothetical case study with 50 employees and an intervention cost of £100, the intervention has a net cost saving of £4,207 per employee (Table 3). This is because the £5,000 cost of the intervention would be offset by a reduction in absenteeism and presenteeism. Because there is uncertainty around model inputs, the exact magnitude of the result should be treated with caution. The results cannot be generalised to all organisations because the inputs will vary by organisation and setting.

**Table 3:** Hypothetical case study results.

	Incremental costs*
Cost of absenteeism	-£40,798
Cost of presenteeism	-£174,575
Intervention cost	£5,000
**Total costs**	**-£210,373**
**Net cost per person**	**-£4,207**

* These results cannot be generalised to all organisations because the inputs will vary by organisation and setting.

### Sensitivity analysis

**Fig 2:**
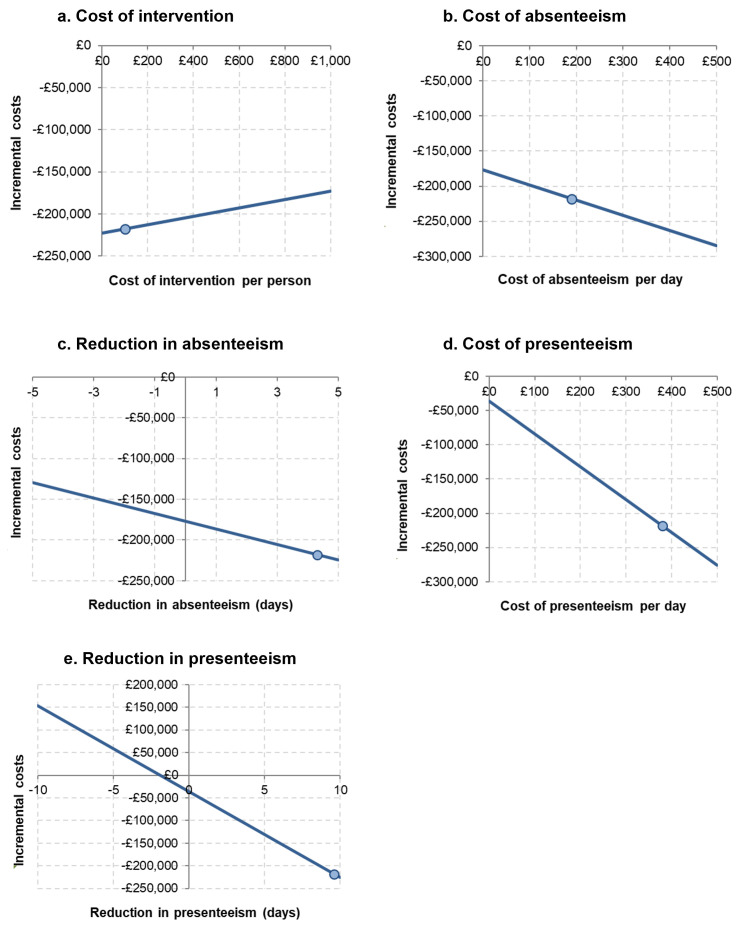
Sensitivity analysis.

Sensitivity analysis assessed the impact of varying each input independently. Fig 2 shows the one-way sensitivity analyses for each variable using the hypothetical case study data. The intervention is more likely to be cost saving when the intervention cost is low and the daily cost of absenteeism and presenteeism are high. Larger reductions in absenteeism and presenteeism also make it more likely to be cost saving.

### Hypothetical scenario analysis

The results of the hypothetical scenario analysis are presented in Table 4. The results demonstrate the need to evaluate the balance between the cost of the intervention and the impact that it has on productivity outcomes. For example, in Scenario 2, the reduction in absenteeism of 0.6 days with no (recorded) impact on presenteeism or staff turnover is not enough to outweigh the intervention cost of £160 per participant. On the other hand, in Scenario 1, the total cost of the intervention (rather than being a per-person cost) and the higher reduction in absenteeism and presenteeism result in a net saving per person of £637. These results demonstrate how the impacts of workplace interventions can vary substantially between settings.

**Table 4:** Hypothetical scenario analysis results.

		Cost
Scenario 1: Large company with high levels of absenteeism and presenteeism	Cost of absenteeism	-£168,480
	Cost of presenteeism	-£324,000
	Intervention cost	£110,000
	**Total costs**	**-£382,480**
	**Net cost per person**	**-£637**
Scenario 2: Medium company with low levels of absenteeism	Cost of absenteeism	-£11,655
	Intervention cost	£12,000
	**Total costs**	**£345**
	**Net cost per person**	**£5**
Scenario 3: Small company with medium levels of absenteeism and staff turnover	Cost of absenteeism	-£40,700
	Cost of staff turnover	-£11,400
	Intervention cost	£30,000
	**Total costs**	**-£22,100**
	**Net cost per person**	**-£221**
Scenario 4: Small company with high levels of staff turnover	Cost of staff turnover	-£7,295
	Intervention cost	£2,080
	**Total costs**	**-£5,215**
	**Net cost per person**	**-£130**
Scenario 5: Micro company with low levels of absenteeism and medium presenteeism	Cost of absenteeism	£222
	Cost of presenteeism	-£1,332
	Intervention cost	£825
	**Total costs**	**-£285**
	**Net cost per person**	**-£57**

## Discussion

This analysis, using a hypothetical case study based on a combination of published evidence and assumptions, demonstrates that mental health interventions at work can be cost saving for an employer in some settings. It is recommended that decision makers make use of the model to understand the potential economic and wellbeing implications when considering the introduction of a new intervention in the workplace.

The key factors that affected the results of this study include the size of the organisation and costs of the intervention, absenteeism, presenteeism, and staff turnover. The analysis also found that an intervention is more likely to be cost saving when the baseline level of absenteeism is high, baseline presenteeism is high, baseline staff turnover is high, the intervention cost is low, and the intervention is demonstrated to have a positive influence on absenteeism, presenteeism or staff turnover.

It has previously been reported that external factors, such as an individual’s personal life and workplace culture, may impact on absenteeism, presenteeism and staff turnover [19–21]. Therefore, external factors may affect the results of this study. For example, reasons for presenteeism could include financial concerns, job insecurity or management responsibilities, a well as the stigma surrounding mental health and sickness absence [20]. It was reported that of two studies with a combined sample of nearly 5,000 people, 21% were more likely to continue working when feeling unwell from mental health problems due to shame or embarrassment, 24% believed their employer would make them come into work if they were not physically unwell, and 42% felt worried or embarrassed about discussing their mental health issue with their employer [20].

It is important to consider that an intervention may be cost saving but might have a negative impact on employee wellbeing outcomes. For example, if an intervention involves substantial training demands on top of an employee’s usual workload, they may experience an increase in stress, exhaustion or reduced wellbeing [22]. A survey also demonstrated the lack of positive wellbeing impact with some interventions, finding no significant difference in wellbeing outcomes between participants and nonparticipants for individual-level interventions, such as relaxation practices, time management, coaching, financial well-being programmes, wellbeing apps, online coaching, sleep apps and sleep events [23]. A qualitative evidence synthesis highlighted key barriers to the implementation of mental health interventions that could reduce their impact, including a lack of leadership or managerial support, a lack of (or minimal) technical support, stigma, and confidentiality issues [24]. Therefore, the economic model alone cannot be used to make a decision on mental health intervention implementation. Other information, such as wellbeing outcomes, stakeholder engagement and employee feedback, should be used in conjunction [25].

The model does not directly capture organisational outcomes such as counterproductive work behaviours, interpersonal conflict, errors, and disengagements. However, these outcomes should be indirectly reflected in the productivity, sickness absence, and/or staff turnover outcomes produced by the model. Therefore, this model should adequately capture the organisational impacts of mental health interventions from the perspective of the employer.

A common method to estimate the productivity costs (i.e., absenteeism and presenteeism) is using a human capital approach that uses gross wage to estimate costs per day [26]. However, alternative approaches, such as friction cost or multiplier methods, should also be considered [27]. A variety of factors may influence productivity costs, including but not limited to, statutory sick pay, employee sick-pay benefits, internal labour reserves, and the ability for employees to make up for lost work [28,29]. In addition, average productivity costs may be influenced by potential variation in the prevalence of absenteeism and presenteeism based on role or salary within an organisation [28].

Implementing mental health interventions in the workplace can have wider benefits across society, such as to the healthcare system and local authorities [30]. These factors are not quantified in the model due to the lack of data to capture these benefits in different settings. However, it can be assumed that where the evidence suggests a positive outcome for the employer and the employee, the societal impact is also likely to be positive. The findings of several systematic literature reviews support this by demonstrating that some mental wellbeing interventions can be cost effective from both an employer and societal perspective [9,31,32].

This analysis contributes to the body of literature on workplace mental health interventions by targeting a potential barrier faced by policy makers and campaigners by demonstrating not just the benefit of these interventions to employees but also the benefit to the employer.

The model is flexible so that organisations can include their own specific inputs. The model will not provide a definitive decision on whether to implement an intervention in the workplace but provides a user-friendly tool to support decision making that can be used in conjunction with other information (https://www.yhec.co.uk/resource/mental-health-at-work-economic-model/).

## Limitations

The baseline inputs have high levels of uncertainty so the magnitude of the result should be treated with caution. For example, the results are driven by the cost savings associated with employer benefits, which are derived from one source and have a high level of uncertainty [15]. The results cannot be generalised to all organisations because the inputs will vary by organisation and setting. Furthermore, it is impossible to draw broad conclusions from the scenarios because there is substantial variability in the interventions available. Instead, the model is flexible, and it is recommended that employers and decision makers use the model to explore the potential economic and wellbeing implications of implementing a mental wellbeing intervention in a specific workplace. Being unable to translate employee wellbeing outcomes into economically meaningful terms means that the model will likely underestimate the full economic benefits of an intervention.

## Conclusions

Mental wellbeing interventions may influence a range of outcomes but outcomes demonstrating a mental wellbeing benefit to employees may be challenging to translate into monetary value. It is not possible to draw broad conclusions from the hypothetical case study and scenarios evaluated in this study because there is variability in the interventions available and heterogeneity in the employment sector. The model described in this paper has previously been used to inform national public health guidance on workplace wellbeing [13]. It is recommended that the model (which is available online) is used by decision makers and employers to understand the potential economic and wellbeing implications when considering the introduction of a new mental wellbeing intervention in the workplace.
